# Nanodispersions of Polyelectrolytes Based on Humic Substances: Isolation, Physico-Chemical Characterization and Evaluation of Biological Activity

**DOI:** 10.3390/pharmaceutics13111954

**Published:** 2021-11-18

**Authors:** Elena V. Uspenskaya, Anton V. Syroeshkin, Tatiana V. Pleteneva, Ilaha V. Kazimova, Tatiana V. Grebennikova, Irina T. Fedyakina, Varvara V. Lebedeva, Oleg E. Latyshev, Olesia V. Eliseeva, Viktor F. Larichev, Timur M. Garaev, Tatiana V. Maximova, Mariya A. Morozova, Pham My Hanh

**Affiliations:** 1 Department of Pharmaceutical and Toxicological Chemistry, Medical Institute, RUDN University, 6 Miklukho-Maklaya Street, 117198 Moscow, Russia; syroeshkin_av@pfur.ru (A.V.S.); pleteneva_tv@pfur.ru (T.V.P.); kazimova.ilaha96@gmail.com (I.V.K.); maximtat@mail.ru (T.V.M.); morozova-ma@rudn.ru (M.A.M.); gaubu27@gmail.com (P.M.H.); 2 National Research Center for Epidemiology and Microbiology Named after the Honorary Academician N. F. Gamaleya, 18 Gamaleya St., 123098 Moscow, Russia; t_grebennikova@mail.ru (T.V.G.); irfed2@mail.ru (I.T.F.); Lebedevavv@ya.ru (V.V.L.); oleglat80@mail.ru (O.E.L.); olesenka80@mail.ru (O.V.E.); vlaritchev@mail.ru (V.F.L.); tmgaraev@gmail.com (T.M.G.)

**Keywords:** polyelectrolytes, nanodispersion, humic and fulvic acids, dried droplet method, particle size measurement, zeta potential, polydispersity antiviral activity, VERO-E6

## Abstract

Natural polyelectrolytes, including in the form of complexes with colloidal particles, are increasingly used in pharmacy due to the possibility of regulated attachment of medicinal substances and their targeted delivery to the target organ. However, the formation, stability, and molecular-mass characteristics of polyelectrolyte nanodispersions (ND) vary depending on the nature and composition of the medium of their origin. This is due to the lack of standardized approaches to quality control and regulatory documentation for most natural ND. In this paper, we first introduced the isolation, followed by investigations into their physico-chemical properties and bioactivity. Using the dried droplet method, we were able to detect the “coffee ring effect”. Fractographic studies of the surface structure of EHA and FA dried samples using SEM showed its heterogeneity and the presence of submicron particles encapsulated in the internal molecular cavities of polyelectrolyte. FTIR spectroscopy revealed the ND chemical structure of benzo-α-pyron and benzo-γ-pyron, consisting of nanoparticles and a branched frame part. The main elements detected by X-ray fluorescence in humic substance extract and fulvic acid include Si, P, S, K, Ca, Mn, Fe, Cu, Zn, whereas Fe is in high concentrations. The UV-spectra and fluorescent radiation demonstrated the possibility of studying the effect of the fulvate chromone structure on its optical properties. It is shown that dilution of the initial solutions of polyelectrolytes 1:10 contributes to the detection of smaller nanoparticles and an increase in the absolute value of the negative ζ-potential as a factor of ND stability. A study of the EHS effect on the SARS-CoV-2 virus infectious titer in the Vero E6 cell showed the effective against virus both in the virucidal scheme (the SI is 11.90–22.43) and treatment/prevention scheme (the SI is 34.85–57.33). We assume that polyelectrolyte ND prevent the binding of the coronavirus spike glycoprotein to the receptor. Taking into account the results obtained, we expect that the developed approach can become unified for the standardization of the ND natural polyelectrolytes complex, which has great prospects for use in pharmacy and medicine as a drug with antiviral activity.

## 1. Introduction

Nanodispersions (ND) are nanoparticle–liquid systems containing particles and agglomerates with a size of 0.1–150 nm [1]. Natural polyelectrolytes are a unique raw material for the development of pharmaceutical products, due to their ability to renew in natural conditions and environmental safety [2]. The most important natural polyelectrolytes that are used in biomedical fields include polysaccharides (algae polysaccharides, pectins), proteins (collagen, gelatin, white blood products, lectins), and natural polyesters [3,4,5,6,7,8,9]. Examples of synthetic organic and inorganic biodegradable polyelectrolytes—a class of experimental adjuvants—are Azoximer bromide (Polyoxidonium), Polyphosphazenes (PPHOs) [10,11,12,13,14,15,16]. The increase in the rate of introduction of polymer compositions for biomedical purposes is facilitated not only by the increasing need for new polymer implants, controlled methods of drug delivery (controlled release systems), but also by the replacement of expensive matrices with more economically affordable ones [17]. Polymer products and medicinal preparations have satisfactory mechanical characteristics, improved solubilization properties, swelling ability and sorption characteristics, which are not characteristic of other groups of biomaterials [18,19,20,21]. The development, research, and production of most polyelectrolyte materials for medical and biological purposes are regulated by the GMP standard [22]. The disadvantage of polyelectrolytes and complexes based on them is their unsatisfactory relative aggregate stability (AS) due to the large interfacial surface and the excessive value of the Gibbs surface energy [23,24,25]. The control of the average particle size of the aqueous dispersion of polyelectrolytes contributes to an increase in the relative aggregate stability of the systems [26,27]. Due to their size (about 100 nm), comparable to the size of cells (10–100 µm), viruses (20–450 nm), proteins (5–50 nm), nanoparticles (colloidal particles), approaching in size to a biological object, can interact and bind with it [28,29].In this connection, when obtaining carriers of medicinal substances, for example, when forming self-organizing nanoparticles of polyelectrolytes with electrostatically complementary surfactants, the attention of researchers is directed to the requirements for the dimensional characteristics of carrier particles and their stabilization [30,31,32]. To stabilize ND, natural or synthetic polymers are used, which are adsorbed on the surface of nanoparticles with the formation of a structural-mechanical barrier that prevents the particles from sticking together [33].

The authors [34,35] suggested the possibility of obtaining ND for medical and biological purposes based on humic substances. Humic substances are complex, heterogeneous, polydisperse mixtures formed in soils, sediments, and natural waters as a result of biochemical reactions during decomposition and transformation of plant and microbial residues (humification) [36]. According to [37], the structure of humic substances as multifunctional compounds does not have a constant chemical composition, which is explained by the stochastic nature of humification processes [38]. Molecules of humic substances of different molecular weights can bind and form a supramolecular humic structure; the degree of aggregation depends on the pH, ionic strength, and mineral composition of the solution [39]. Humic substances belong to anionic polyelectrolytes since they contain polar functional groups in their structure, such as carboxyl, hydroxyl (alcohol, phenolic) ones, that are able to dissociate with the formation of negatively charged particles. The ability of HS to occlude metals determines the content of micronutrients in their solutions in bioavailable forms, as well as the plant metabolism. In recent decades, there have appeared numerous publications about studies of the pharmacological activity of HS and FA against human immunodeficiency virus HIV-1 [40], influenza virus [41], herpes simplex virus-1 (HSV-1) [42], tick-borne encephalitis virus (TBEV) [43], bacteria Enterococcus faecalis and Klebsiella pneumoniae [44], and phytopathogenic fungi [45]. The authors demonstrated much higher activity of the humic acids versus fulvic acids: HA > HMA > FA, as well as the dependence on the source of the humic acid release: coal > peloid > peat. The significant role of active components—aliphatic fragments (for HA) and COOH, OH fragments (for FA), which determine a positive correlation with the inhibition rates of HAs and FAs against phytopathogenic fungi, is discussed [46]. Review publications describe the possible mechanisms of HS and FA effect in preventing the replication of the SARS CoV-2 virus by sorption on the virus envelope protein, thereby blocking the sorption of viral particles on the cell surface [47]. Here, there are currently no drugs that have shown clear and consistent benefits in treating SARS CoV-2, but numerous trials in different countries are underway, suggesting that HS may reduce symptoms. 

All these data create prerequisites for the creation of new biologically active substances based on natural polyelectrolytes for the pharmaceutical industry. In this study, we sought to develop approaches to the isolation and study of the physico-chemical properties of natural polyelectrolytes. In this paper, we first introduced the isolation, followed by investigations of their physicochemical properties and bioactivity. We expect that the developed protocol, including, inter alia, the study of the virucidal activity of HS nanodispersions, will increase the effectiveness, safety and overall quality of a pharmaceutical product based on an affordable and low-toxic natural polymer to develop a promising drug.

## 2. Materials and Methods

### 2.1. Reagents

#### 2.1.1. EHS and FA Samples

A natural complex of humic-fulvic acids isolated from low-lying peat, sapropel and some varieties of brown coal (leonardite) using the technology of the VimaVita Company (Sistema-Biotechnologies, Moscow, Russian Federation). The HS concentrate containing humic acids (HA), himatomelanic acids (HMC), fulvic acids (FA) and structural analogs of humic substances was obtained by the method of oxidative-hydrolytic destruction of lignin-containing raw materials (solid-phase fermentation) with subsequent purification [48]. As a result of high-intensity acoustic cleaning, a concentrated viscous colloidal disperse system of humic substances of dark brown color was obtained for research (pH = 7.98 ± 0.1).

Fulvic acid extract (Terra Aquatica, Paris, France) in the form of a liquid, transparent solution of brown color, obtained by extraction from a special type of brown coal – leonardite (pH = 5.77 ± 0,1). To study the physico-chemical properties of HS and FA, aqueous solutions of the initial concentrate were prepared. All solutions of humic substances fractions were stored at +4 °C. Test samples for in vitro studying with Vero were dissolved in the medium (DMEM + glutamine (292 µg mL^−1^) (Capricorn Scientific GmbH, Ebsdorfergrund, Germany) + penicillin/streptomycin (50 units/mL/50 µg mL^−1^) + inactivated fetal bovine serum, 2% (FBS) (Biosera, Kansas City, MO, USA) to obtain a final dilution of 1/800. The dry total residue after complete vaporization of the EHS substance was 7.34−10^−2^ g mL^−1^, correspondingly the initial concentration obtained was 0.091 mg/mL. Then, a concentration of 0.091–0.003 mg mL^−1^ was obtained by successive twofold dilution of the substance in the wells of the test plate when mixed with the viral suspension.

#### 2.1.2. Vero-E6 Cell Culture

The study was carried out on a finite kidney cell line of the African green monkey (Chlorocebus aethiops) Vero-E6. MEM medium with glutamine containing 10% and 2% FBS, respectively and gentamicin (50 μg mL^−1^) was used as a growth medium (GM) for growing cells and as a supporting medium (SM) for setting up a reaction. Vero-E6 cells were cultured in 96-well plates in a volume of 100 μL of GM for 24 h at 37 °C in an atmosphere with 5% CO_2_. The inoculation dose was 18,000 cells/well.

#### 2.1.3. Virus

The study used the SARS-CoV-2 human coronavirus, passage 3, with infectivity of 10^7.5^ TCID _50_/mL. Strain description: hCoV-19/Russia/Moscow-PMVL-12/2020 (EPI_ISL_572398) GISAD: PMVL-12. Booking reference EPL_ISL_572398. 

#### 2.1.4. Hydroxychloroquine Sulfate (HDR)

HDR (2-[[4-[(7-Chloro-4-quinolinyl)amino]pentyl]ethylamino]ethanol sulfate) an anti-inflammatory, antimalarial and antirheumatic agent, inhibits Toll-like receptor 9—commercially available substance (purity > 99% Promochem, Oulu, Finland). It was used as a reference drug. HDR was dissolved in the same medium according to the test concentrations of 0.013 and 0.04 mg mL^−1^.

### 2.2. Evaporation of Colloidal Nanodispersions-Dried Droplet Method (DDM)

To study polymer-colloidal polyelectrolytes, sedimentary structures were obtained after the evaporation and heat treatment of colloids. The DDM is a widely used sample pre-treatment in analytical chemistry that involves placing a droplet of solution onto the substrate and drying for analytical testing. The determination was carried out according to the requirements [49]. For this purpose, the accurately measured weight of the tested liquid substance, pre-dried and brought to constant weight, was placed in an evaporating dish (porcelain). The drying was carried out at 105 ± 5 °C for 6 h in a drying oven BINDER FD (Hielkema Testequipment B.V., Tuttlingen, Germany) that provides uniform heat treatment of the entire usable chamber volume [50]. The weight of the sample dish was determined and recorded every hour by removing the dish from the oven and allowing it to cool at room temperature in a desiccator for 30 min. The loss on drying (%) was calculated according to the following equation:

(1) $$w=\frac{m_{2}-m_{3}}{m_{2}-m_{1}}\cdot 100\%,$$

where *m*_1_ is the weight of the measuring cup brought to a constant weight (g); *m*_2_ is the weight of the measuring cup containing the tested sample before drying (g); *m*_3_ is the weight of the measuring cup containing the tested sample after drying (g).

### 2.3. Scanning Electron Microscopy (SEM)

The morphology of polyelectrolytes particles was characterized using a fourth generation scanning electron microscope Mira 3 (Tescan, Brno, Czech Republic) with a Schottky cathode with a maximum resolution of 1 nm and a maximum increase of 1,000,000. The EHS and FA samples dried to a constant mass were evacuated and fixed to tables in the SEM chamber on a double-sided conductive carbon tape. The “charging” effect was realized by spraying a thin layer of conductive material, carbon, onto the surface of the powder samples [51].

### 2.4. Fourier Transform Infrared (FT-IR) Spectroscopy 

To obtain and analyze the vibrational spectra of the EHS and FA samples in the spectral range from 4000 to 750 cm^−1^, an IR Fourier spectrophotometer Agilent Cary 630 (Agilent, Santa Clara, CA, USA) with a transmission attachment was used [52]. Sample preparation—solid residue after drying—for spectrum recording was carried out following the requirements [53]. For this, about 1 mg of dry residue was triturated with 400 mg of carefully ground and dried potassium bromide until uniform state and compressed for 3–5 min to obtain a disk diameter of about 13 mm to have a spectrum of suitable intensity. 

### 2.5. X-ray Fluorescence (XRF)

An energy dispersive X-ray fluorescence spectrometer (EDX-7000P, Shimadzu Europa GmbH, Duisburg, Germany) based on a silicon drift detector with thermoelectric cooling equipped with the PCEDX-Navi software (Shimadzu Europa GmbH, Duisburg, Germany) package was used to carry out the non-destructive elemental composition of powder and liquid EHS and FA samples. The range of elements measured by the X-ray fluorescence method is from 11Na to 92U; the X-ray generator is a tube with Rh-anode, current 1–1000 µA; the irradiated area controlled by the collimator was 10 mm [54]. Pelleted powder or liquid sample of EHS and FA was placed in a closed cuvette covered with a mylar (lavsan) film in an air atmosphere and placed exactly in the center of the instrument window. The intensity of the secondary fluorescent radiation was measured to determine the elemental composition of the sample. The study time was 50 s for each element (group).

### 2.6. Fluorescence and UV-Spectroscopy

To obtain the fluorescence spectra of a series of dilutions of the EHS and FA samples, we used an AGILENT Cary Eclipse spectrofluorimeter (Agilent, Santa Clara, CA, USA) with two ultrafast scanning monochromators. The excitation wavelength was 280 nm. The fluorescence spectra in the range from 300 to 800 nm with the maxima of violet and green fluorescence were studied.

The absorption spectrum of FA dilute aqueous was obtained in the range from 200 nm to 350 nm using AGILENT Cary 60 equipment (Agilent, Santa Clara, CA, USA).

### 2.7. Dynamic Light Scattering (DLS)

A Zetasizer Nano ZSP (Malvern Panalytical, Worcestershire, UK) based on dynamic light scattering (DLS) was used to measure the size of nanoparticles in the EHS and FA samples [55]. For this purpose, aqueous dispersions of colloidal polyelectrolyte nanoparticles with concentrations from 3·10^−^^4^ to 7·10^−^^5^ g·mL^−^^1^ for HS and FA, respectively, were prepared. Laser Doppler Micro-electrophoresis was used to measure zeta potential based on determining the velocity of nanoparticles while they are moving due to electrophoresis.

### 2.8. Statistical 

An analysis of all data were acquired from *n* ≥ 3 independent experiments and are presented as the mean ± standard deviation (SD). Statistical analyses were conducted using Microsoft Excel 5.0 и GraphPad Prism 6.01. in which comparisons for one condition between two groups were performed by Student’s t-test with a significance level of *p* < 0.05 throughout the study.

## 3. Results and Discussion

### 3.1. Evaporation of Colloidal Nanodispersions-Dried Droplet Method (DDM)

**Figure 1:**
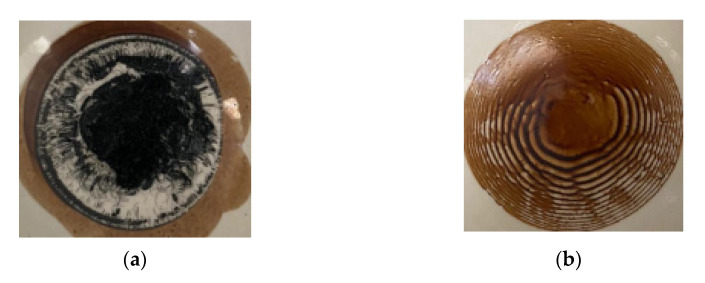
Dried drops (“coffee ring effect”) of the investigated colloidal dispersions: (**a**) extract of humic substance; (**b**) fulvic acid.

Evaporation of the limited volumes of the studied colloidal nanodispersions allowed us to detect ring-type structures. During the droplet drying process, the solutes would converge at the droplet edge and cause inhomogeneous solutes distribution (“coffee ring effect”, CRE) [56,57,58] (Figure 1). 

The resultant ring patterns can be simply observed with the naked eye. The induced Marangoni flow would reversibly transport the particles at the periphery toward the centerline. The spatial patterns of the microbeads were characterized, showing the dense packing of the microbeads at the center of the droplet and thinning of the ring pattern at the periphery [59]. Temperature is an important factor influencing self-assembly in a drying drop of solution, and, consequently, controlling the architecture of ensembles of micro- and nanoparticles [60,61]. At the final stage of evaporation processes, very diverse morphological structures arise. 

#### 3.1.1. The Structure of the Polyelectrolyte’s Particles

**Figure 2:**
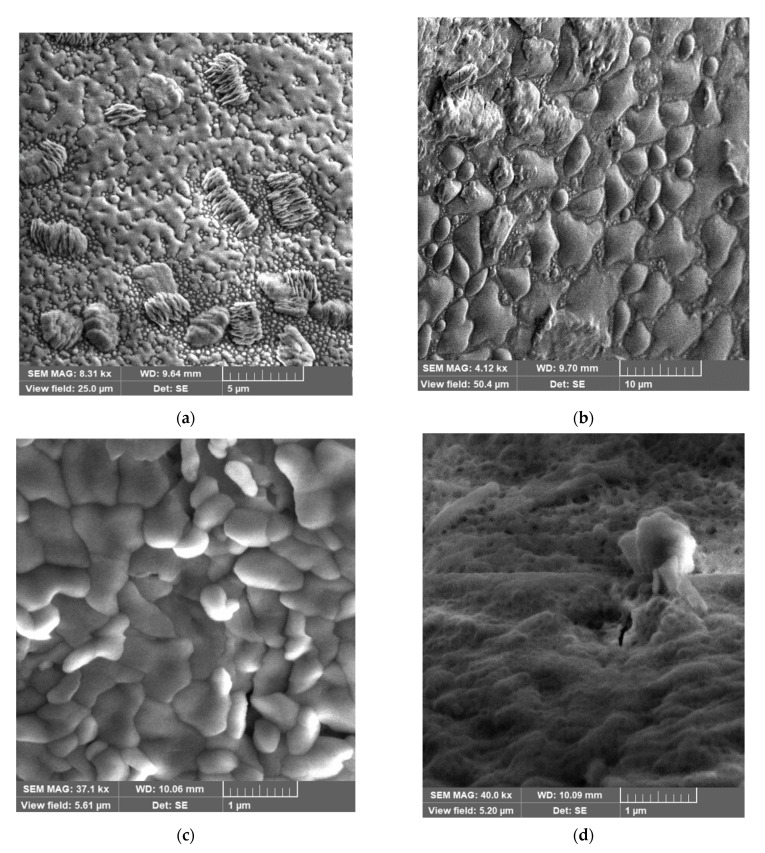
Morphology of sedimentary structures by SEM image with high mag.: (**a**,**b**) extract of humic substance; (**c**,**d**) fulvic acid.

Visualization of the complex ordered morphology of the near-surface layer requires additional advanced microscopy methods. The SEM images of the near-surface layer in dried samples of EHS, FA are presented on Figure 2.

SEM images of the dried samples of EHS and FA with a high spatial resolution and depth of field in secondary (SE) electrons allow for fractographic studies of the surface structure, defects, and fractures [62]. The surface structure of solid particles of EHS is heterogeneous and is represented by submicron particles encapsulated in the internal molecular cavities of polyelectrolyte. The morphology of the FA surface structure becomes more homogeneous, with signs of adhesion between particles.

#### 3.1.2. Determination of Dry Residue

By Table 1 data, mass loss and residue after drying were determined in the HS and FA samples under study.

The determination of the dry residue is 7.34·× 10^−2^ g mL^−1^ and 3.36·× 10^−3^ g mL^−1^, respectively for EHS and FA. Additionally, the weight loss due to water and volatile substances is 92.63% and 99.66%. The data obtained allow us to determine the concentration of the studied dilutions of ND polyelectrolytes. 

### 3.2. Spectroscopic Methods for the Study of HS and FA Qualitative and Quantitative Composition

#### FT-IR Spectroscopy Identifying 

**Figure 3:**
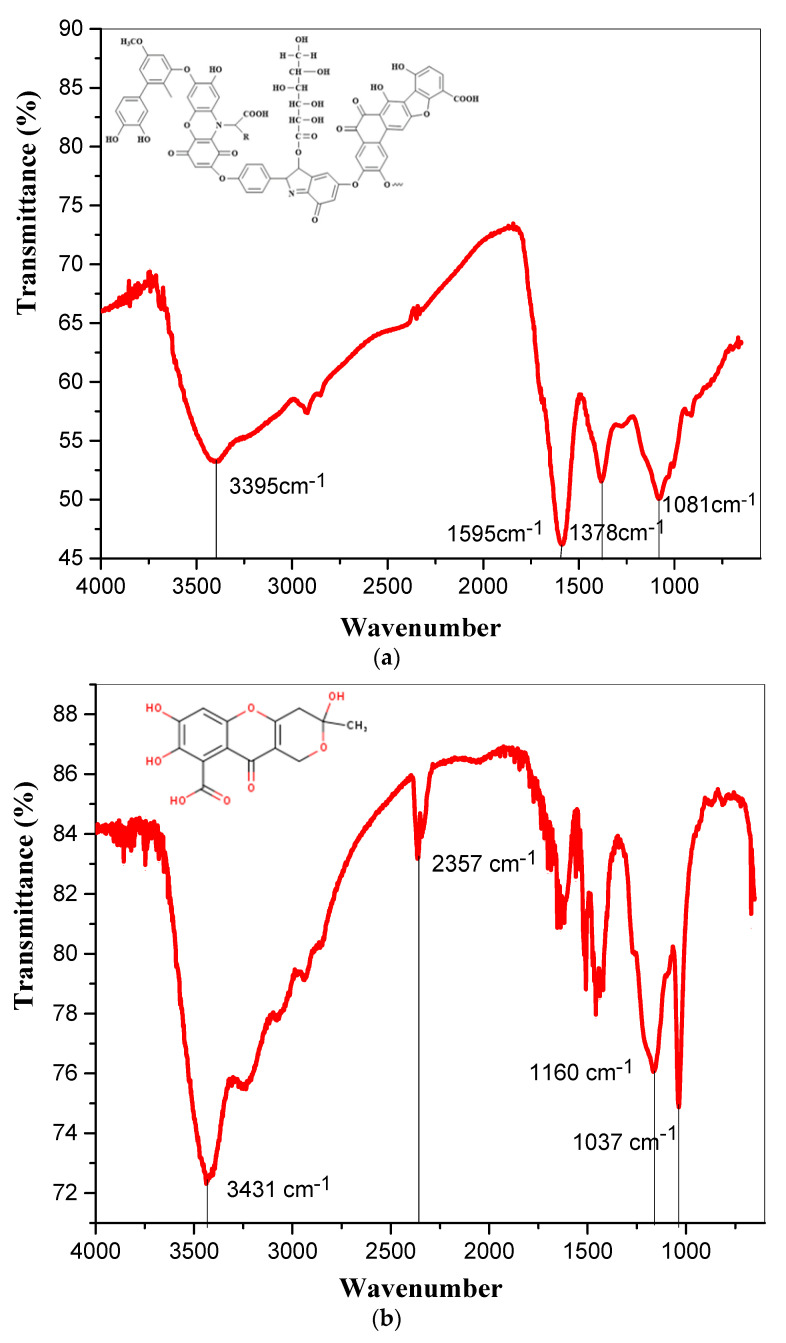
FT-IR spectrum of (**a**) extract humic substances; (**b**) fulvic acid sample.

The identification of the studied high-molecular samples to determine their belonging to organic aromatic hydroxycarboxylic acids was carried out by comparing their unique “molecular spectral imprint” with the characteristic frequencies of bond fluctuations in functional groups [63]. The vibrational-rotational spectra of the EHS and FA samples obtained by the disk technique with potassium bromide are shown in Figure 3.

The EHS spectrum is represented by several characteristic bands at 3395, 1595, 1378, and 1081 cm^−1^. In a comparative analysis of the transmission spectra of the compounds under investigation it was found that the EHS sample is characterized by higher values of the extinction coefficients, as a result of which the percentage of light transmission is noticeably reduced, which is probably caused by the nonstoichiometric composition and irregular heterogeneous structure with numerous functional groups. Stretching vibrations of free and bound hydroxyl groups (OH) usually form a broadband region in the frequency range from 3200–3670 cm^–1^ [64]. However, due to the coordinating influence of iron atoms present in the EHS composition and the formation of complex nanostructures containing Me-O bonds, the vibrations of the O-H bond can be shifted to the low-frequency region (≈3400 cm^–1^). In addition, the stretching vibrations of primary or associated amino groups and, probably, imines, can also be attributed to the absorption band at ≈3400 cm^–1^. The presence of a carbon skeleton manifests itself as a band of stretching vibrations of the C–H bond at 2900 and 2850 cm^–1^ [65] (Table 2).

The obtained vibrational-rotational spectra of EHS and FA are characterized by similar vibration frequencies and the shape of the passbands for the derivatives of benzo-pyrone and its secondary metabolites called benzo-α-pyrone (coumarin) and benzo-γ-pyrone (chromone) due to the condensation of pyron derivatives with benzene in plants [66,67,68].

### 3.3. Elemental Analysis

**Figure 4:**
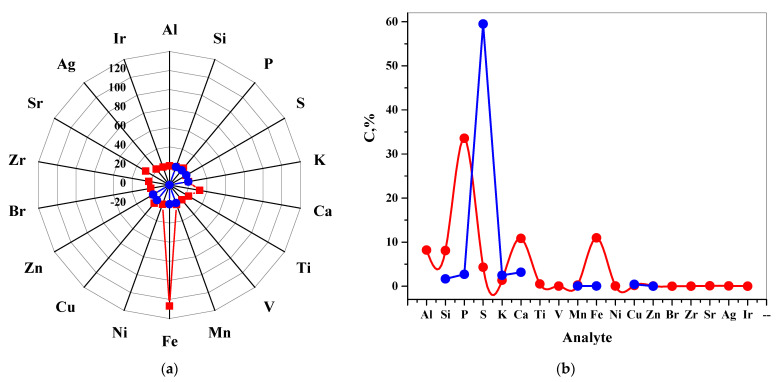
X-Ray fluorescence analysis of the elements in the dry residue of EHS (red) and FA (blue) samples: (**a**) intensity dependence; (**b**) dependence on element fraction.

The elemental contents and the phytochemical components of humic acids are crucial for their medical purposes. Therefore, the variations of the elemental composition of EHS and FA have been investigated using a non-destructive elemental analysis technique (Figure 4).

As can be seen in Figure 4, atoms of the elements Si, P, S, K, Ca, Mn, Fe, Cu, Zn were found in both EHS and FA samples. Noteworthy is the high intensity of the X-ray fluorescence signal for Fe atoms in the EHS sample. It is known that humic substances, participating in the formation of chelates with iron, contribute to plant nutrition [69]. Depending on the solubility and molecular size of HS, humified fractions of organic matter in soil sediments contribute to the creation of an Fe reservoir available to plants [70]. It has been shown [71,72] that the distribution and release of Fe within plants can be controlled if they are supplied with water-soluble Fe-HS complexes in comparison with other natural or synthetic chelates. In the case of Fe, highly stable HS complexes mainly include O-containing groups (carboxyl and phenolic) [73]. It is considered [74] that polyelectrolyte macromolecules form a shell around particles in nanodispersions-polyelectrolyte complexes of the “macromolecule-nanoparticle” type, prevent further growth of metal particles and aggregation [75]. Fulvic acids are less prone to the formation of insoluble complexes with metals.

#### Study of Fluorescence and Ultraviolet Spectra

**Figure 5:**
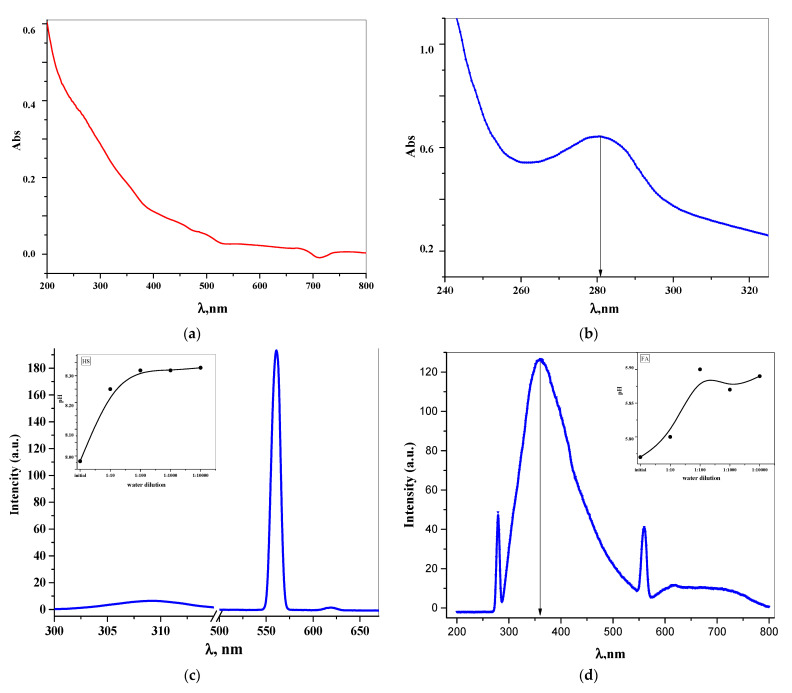
Ultraviolet-visible and fluorescence spectrum at the excitation wavelength of 280 nm: (**a**,**c**) extract humic substances sample water solution (7.3 × 10^−12^%); (**b**,**d**) fulvic acid sample water solution (3.4 × 10^−3^%). pH Dependence of dilutions are shown in the inserts.

It is well known that compounds, which are concentrated benzene nuclei with oxidized pyran (coumarins, chromones), are fluorophores or chromophores due to their high photostability, large Stokes shift, and intense fluorescence with a high quantum yield [76,77]. The fluorescence spectra, which are a tool for investigating the effect of the fulvate chromone structure on its optical properties, were recorded at their excitation wavelength. The electronic absorption and fluorescence emission spectra of aqueous dilution of the FA and EHS liquid samples are shown in Figure 5.

One of the most important properties of humic substances that make up dissolved organic natural carbons (DONC) is fluorescence. Humic-like fluorescence, as the most important characteristic of DONC, is manifested at the ratio λexλem= 280−350360−690  [78]. Samples FA and EHS have maxima of humic fluorescence at λex1λem1 =  280560 (Figure 5). 

The true FA fluorescence spectrum was recorded in the range from 300 to 400 nm, at an excitation wavelength of 280 ± 2 nm, which is associated with the presence of a chromone heterocyclic nucleus in the structure. As a small organic fluorophore, containing several condensed nuclei in a fluorescent open form, the fulvate molecule produces characteristic intense violet fluorescence at 360 nm wavelength [79]. These emission peaks of the FL spectrum can be also caused by excited-state intramolecular proton transfer (ESIPT), which is characteristic of fragments with intramolecular hydrogen bonds [80].

Since the EHS solution demonstrated near-ultraviolet transparency and increased far-ultraviolet absorption in the absence of detectable analytical wavelengths, it is convenient to characterize the EHS structure by fluorescence spectra. The photophysical properties of HS containing numerous closed cycles, variations of substitutes, and delocalized π-bonds have undergone some changes; the fluorescence intensity of EHS appeared in the 620 nm and it significantly increased in the 560 nm region.

### 3.4. Particle Size Measurement and Zeta Potential Control in Nanodispersions of Polyelectrolytes by the DLS

**Figure 6:**
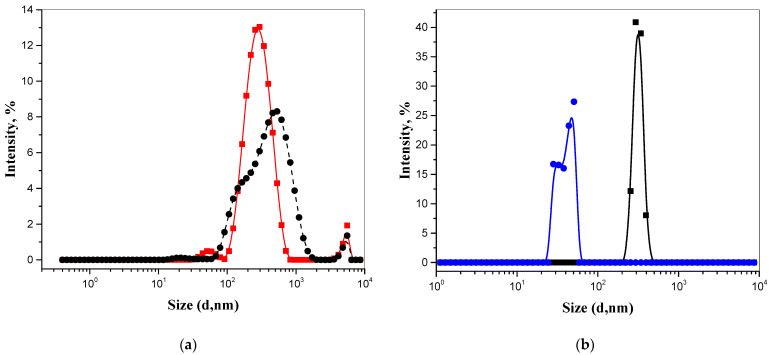
Particle size distribution in nanodispersions of polyelectrolytes: (**a**) EHS: red—7.3 × 10^−5^ g mL^−1^; black—3.71 × 10^−4^ g mL^−1^; (**b**) FA: blu—3.4 × 10^−5^ g mL^−1^; black—3.4 × 10^−4^ g mL^−1^.

Metrological characterization of particles in the studied dispersions of natural polyelectrolytes was carried out by the inverse method of determining the hydrodynamic radius. To characterize nanodisperse systems and describe the properties of particles, the function (I, %-d, nm) and the width (PDl index) of the particle size distribution was used, as well as the ζ-potential as an indicator of the surface charge of particles and a measure of electrostatic interaction (Figure 6 and Table 3). Since the particle size in the dispersed system depends on the concentration [81], the study was carried out with solutions of different dilutions.

Based on the analysis, water dilution of polyelectrolyte solutions 1:10 demonstrates the shift of the maximum distribution on the curve I, %-d, nm to the left, which is especially noticeable in the example of fulvic acid [82]. The size distributions in the submicron region in solutions of both polyelectrolytes are unimodal wide and, consequently, polydisperse, based on the value of the PDl: the values of the polydispersity index range from 0.38 to 1.00 and depend on both the nature of the polyelectrolyte and the concentration. When diluting the FA solution, not only a decrease in the diameter of the particles is observed, but also an increase in the absolute value of the negative ζ-potential. This may be due to an increase in the thickness of the double electric layer as a result of a decrease in the concentration of counterions in the diffusion layer. According to the Deryagin-Landau-Verwey-Overbeck (DLVO) theory, the thickness of the diffuse double electric layer and the surface potential of the particles are the most important factors for the stability of colloids [83]. Knowledge of the zeta potential of the particles in the formulation can be used to logically select the chemical composition of the formulation to select the most suitable materials to ensure stability and increase shelf life.

### 3.5. Biological Activity Studies Using Vero-E6 Cell Culture

Evaluation of the EHS potential effect on the morphology and viability of transplanted cells was carried out in vitro using the Vero-E6 cell line, which demonstrated high sensitivity to viruses, including SARS-CoV-2.

Vero-E6 cells were cultured in 96-well plates in a volume of 100 μL of GM for 24 h and incubated at 37 °C in a humidified 5% CO_2_. The inoculation dose was 18,000 cells/well. After 24 h incubation, double dilutions of the study objects were transferred in 100.0 μl into the wells. After 96 h, the culture medium was removed and 100 μl of GM and 20 μl of vital dye (MTS) (CellTiter 96^®^ AQueous One Solution Cell Proliferation Assay, Promega, G3582, Waltham, MA, USA) were added to each well. After incubation for 3 h at 37 ± 0.5 °C, the results were recorded on a BIO-RAD reader at a wavelength of 490 nm, the reference wave was 630 nm. The concentration of the test substance, which reduces the optical density value by 50% compared to the control cells, was taken as the 50% cytotoxic dose (CC_50_) (Table 4).

Thus, the tabular results indicate the low toxicity of the EHS series on the Vero E6 cell culture.

The study of the direct virucidal, therapeutic, and preventive effect of EHS against SARS-CoV-2 was carried out in a concentration range that is not toxic to the Vero E6 cells. The study used the SARS-CoV-2 human coronavirus. When studying the virucidal effect, the selected dilutions of the drugs (according to CC_50_) were mixed with dilutions of the virus (from 10^−1^ to 10^−7^) in equal volumes of 100 μL and incubated for 1 h at 37 ± 0.5 °C in the atmosphere with 5% CO_2_ and transferred to a plate with a monolayer of washed cells. To study the antiviral activity according to the therapeutic-prevention model scheme, working dilutions of the study objects were added to the cells 1 h before infection with the virus. As a control, we used SARS-CoV-2 virus dilutions without adding the study objects. Each sample concentration was tested in four parallel rows of plate wells. 

The antiviral activity was assessed visually under a microscope 96 h after infection by inhibition of the cytopathic action (CPA) of the virus on the cells. The result was assessed by Δlg_max_—the maximum decrease in the value of the infectious viral dose in the experiment in comparison with the control expressed in decimal logarithms. Each EHS series were tested at six concentrations (Table 5).

Experiments have shown the concentration dependence of the antiviral efficacy of EHS, which may be the result of multivalent interactions between a polyelectrolyte complex and a viral particle [84]. We assume that super branched polyelectrolyte with a high molecular weight can act as a “bait”. This may give additional interaction points in 3D space and the possibility of multipoint binding to the SARS-CoV-2 S-protein [85,86,87,88].

The results obtained allowed determining the IC_50_ value. The results of the cytotoxic and antiviral activities of EHS against the SARS-CoV-2 virus in the Vero E6 cell are shown in Table 6.

A study of the EHS effect on the SARS-CoV-2 virus infectious titer in the Vero E6 cell showed that all EHS series are effective against viruses, both in the virucidal scheme treatment, and prevention scheme. The effective dose is in the range of 0.023–0.041 mg/mL and the suppression of the infectious titer is in the range of 2.82–3.63 lg in the virucidal regimen. In this case, the selectivity index (SI) is 11.90–22.43.

With the treatment and prevention model scheme, the effective dose is significantly lower for the EHS series. Due to this, SI is significantly higher and is equal to 34.85–57.33. 

The results of the antiviral activity study of EHS nanoparticles showed that the samples in doses non-toxic for Vero E6 cells statistically significantly suppressed the reproduction of the SARS-CoV-2 virus. The drugs demonstrate low toxicity, and the SI is quite high. 

Probably, the mechanism of action is realized at the cellular level by analogy with natural fucoidans by preventing the binding of receptors to the coronavirus spike glycoprotein (S-protein) and subsequent penetration into cells through the plasma membrane or through endocytosis [89,90,91,92,93].

## 4. Conclusions

We have studied natural polyelectrolytes, the source of which is humus. This fact causes a significant impact on the structure and properties of natural polyelectrolytes and, as a consequence, the complexity of standardization, interpretation of analytical results and the absence of preclinical and clinical trials. However, numerous reports on the biological activity of humic substances, which have formed as a biochemical form of plant adaptation, create prerequisites for studying their composition, properties, and analytical approaches to quality control. The results of our research suggest that the chemical structure of natural humus polyelectrolytes is due to the stabilizing interaction of macromolecules with nanoparticles of lyophobic sols–metals with the formation of complexes of the macromolecule-nanoparticle type. This is evidenced by the results obtained by X-ray Fluorescence, FT-IR Spectroscopy and DLS methods. For the first time, we were able to detect a clear coffee ring effect during the evaporation of colloidal droplets of the FA sample, which may be the beginning of applied thermohydrodynamic studies in the pharmaceutical analysis of natural polyelectrolytes. Analysis of the morphological structure of dried solid samples by the SEM method allows us to assess their relative heterogeneity, the shape and size of submicron particles. The intensity of the fluorescence signal showed that in addition to the organic structure of the non-stoichiometric composition and irregular heterogeneous structure with numerous functional groups, occluded metal nanoparticles are present in the components of humic acids. Polyelectrolytes modified in this way are a unique transport object for medicinal substances. In addition, our studies have demonstrated the possibility of independent binding of naturally branched polyelectrolyte complexes of humic substances with SARS-CoV-2 S-protein in vitro. We suppose that the proposed approach will be useful in laboratory and clinical settings for the development of standard quality control protocols, as well as the creation of a promising standardized drug with viricidal treatments for the prevention of coronavirus infection.

## Figures and Tables

**Table 1:** Determination of weight loss on drying the samples of the extract of humic substances and fulvic acid.

Extract Humic Substances (EHS)				
t, h	m_1_, g	m_2_, g	m (_tested liquid substance_) g	Loss on Drying (LOD), %
0		127.2238	8.6234	
1		119.2702	0.6698	
2		119.2473	0.6469	
3		119.2411	0.6407	
		**m_3_, g**		
4		119.2362	0.6358	
5		119.2362	0.6358	
6	118.6004	119.2362	0.6358	
				92.63
**Fulvic Acid (FA)**				
**t, h**	**m_1_, g**	**m_2_, g**	**m (_tested liquid substance_) g**	**Loss on Drying (LOD), %**
0		120.1194	4.7984	
1		115.3398	0.0188	
2		115.3391	0.0181	
3		115.3387	0.0177	
		**m_3_, g**		
4		115.3371	0.0161	
5		115.3371	0.0161	
6	115.3210	115.3371	0.0161	
				99.66

**Table 2:** The main transmittance bands in the FT-IR spectrum of EHS and FA.

Frequency Range, cm^−1^	Group	Compound Class	Appearance/ Comments
Humic Substances			
3550–3200	O-H stretching	alcohol	strong, broad/
2920–2850	C-H stretching	alkane	medium/ bands due to Fe nanospheres [65]
1650–1580	N-H bending	amine	medium
1690–1640	C=O stretching	conjugated ketone	strong
1390–1310	O-H bending	phenol	medium
1085–1050	C-O stretching	primary alcohol	strong
Fulvic Acid			
3550–3200	O-H stretching	alcohol	strong, broad intermolecular bonded
3100–3000	C-H stretching	alkene	medium
2920–2850	C-H stretching	alkane	medium/ bands due to Fe nanospheres
2275–2250	N-C=O stretching	amide	strong, broad
1650–1566	C=C stretching	cyclic alkene	medium
1440–1395	O-H bending	carboxylic acid	medium
1450	C-H bending	alkane	methyl group
1275–1200	C-O stretching	alkyl aryl ether	strong
1085–1050	C-O stretching	primary alcohol	strong

**Table 3:** Characterization of nanoparticles in polyelectrolyte dispersions.

Sample	Size ± SD, nm	PDl ± SD	$\bar{\xi \;}$ ± SD, mV	C, g mL^−^^1^
HS	348 ± 230	0.39 ± 0.01	−36 ± 8	3.7 × 10^−4^
	304 ± 117	0.38 ± 0.07	−31 ± 12	7.3 × 10^−5^
FA	280 ± 19	0.94 ± 0.07	−34 ± 11	3.4 × 10^−4^
	50 ± 3	1.00 ± 0	−41 ± 8	3.4 × 10^−5^

**Table 4:** The cytotoxic concentration of EHS against the Vero-E6 cell line.

EHS Test Sample *	CC_50_, mg/mL ± SD
Series 1_2006996	0.516 ± 0.021
Series 2_2006997	0.488 ± 0.018
Series 3_2006998	0.486 ± 0.014

* Since EHS are naturally non-homogeneous biotransformation products, it was important to evaluate the homogeneity of the biological action of a substance from different batches produced by the same manufacturer.

**Table 5:** The effect of EHS on the replication of SARS-CoV-2 in Vero E6 cells.

EHS Test Sample	C, mg/mL	Δlg_max_ ± SD
Series 1_2006996	Virucidal administration regimen	
	0.091	3.19 ± 0.43
	0.046	2.19 ± 0.43
	0.023	0.50 ± 0.71
	0.011	0
	0.006	0
	0.003	0
	Therapeutic and prevention scheme of administration (1 h before infection)	
	0.089	3.19 ± 0.45
	0.044	3.12 ± 0.88
	0.022	1.86 ± 0.18
	0.011	0.5 ± 0
	0.006	0
	0.003	0
Series 2_2006997	Virucidal administration regimen	
	0.091	2.82 ± 0.97
	0.046	1.38 ± 0.53
	0.023	0
	0.011	0
	0.006	0
	0.003	0
	Therapeutic and prevention scheme of administration (1 h before infection)	
	0.091	3.06 ± 0.27
	0.046	2.63 ± 0.18
	0.023	1.31 ± 0.63
	0.011	0.13 ± 0.18
	0.006	0
	0.003	0
Series 3_2006998	Virucidal administration regimen	
	0.091	3.63 ± 0.18
	0.046	1.75 ± 0
	0.023	0
	0.011	0
	0.006	0
	0.003	0
	Therapeutic and *p* prevention scheme of administration (1 h before infection)	
	0.091	3.06 ± 0.26
	0.046	2.75 ± 0
	0.023	1.50 ± 0.25
	0.011	0.3 ± 0.18
	0.006	0
	0.003	0
Hydroxychloroquin sulfate	0.013	5.46 ± 0.29
	0.004	1.21 ± 0.06

**Table 6:** Consolidated data on cytotoxic and antiviral activity EHS.

Test Sample	CC_50_, mg/mL	Antiviral Activities against the SARS-CoV-2 Virus		
		Δlg_max_	IC_50_, mg/mL	SI
Virucidal administration regimen				
Series 1_2006996	0.516	3.19	0.023	22.43
Series 2_2006997	0.488	2.82	0.041	11.90
Series 3_2006998	0.486	3.63	0.024	20.25
Treatment and prevention model scheme				
Series 1_2006996	0.516	3.19	0.009	57.33
Series 2_2006997	0.488	3.06	0.014	34.85
Series 3_2006998	0.486	3.06	0.013	37.38

## Abbreviations

COVID-19	Corona Virus Disease 2019
CRE	coffee ring effect
CC_50_	half maximal cytotoxic concentration
CPA	cytopathic action
DLVO	Derjaguin-Landau-Verwey-Overbeck theory
DONC	dissolved organic natural carbons
DLS	dynamic light scattering
DDM	dried droplet method
EHS	extract humic substances
ESIPT	excited state intramolecular proton transfer
EDXRF	energy dispersive X-ray fluorescence
FA	fluvic acids
FBS	fetal bovine serum
FRO	ferric chelate reductase
GM	growth medium
GPM	good manufacturing practice
HA	humic acids
HS	humic substances
HMA	hymatomelanic acid
HDR	hydroxychloroquine sulfate
IC_50_	half maximal inhibitory concentration
IHS	insoluble HS
HIV-1	human immunodeficiency virus
HSV-1	simplex virus-1
PDI	polydispersity index
IRT	iron transporter
IR	infra-red
MEM	minimum essential medium (alpha 1X)
MTS	vital dye (1-solution methyl thiazolyl tetrazolium)
ND	nanodispersions
SE	supportive environment
SEM	scanning electron microscopy
SI	selectivity index
SARS-CoV-2	severe acute respiratory syndrome-related coronavirus 2
SLS	static light scattering
SM	supporting medium
Spirotox test	Spirostomum Ambiguum Acute Toxicity Test
TBEV	tick-borne encephalitis virus
TCID50	50% tissue culture infectious dose
WEHS	water extractable humic substances
Vero-E6	cell culture derived from epithelial buds of African green monkey and transfected with a viral gene
